# Development of a Novel Friction Model for Machining Simulations in Unidirectional Composite Materials

**DOI:** 10.3390/polym14050847

**Published:** 2022-02-22

**Authors:** Oscar Seward, Fernando Cepero-Mejías, J. Patrick A. Fairclough, Kevin Kerrigan

**Affiliations:** 1 AMRC with Boeing, Advanced Manufacturing Park, Wallis Way, Catcliff, Rotherham S60 5TZ, UK; f.cepero@sheffield.ac.uk (F.C.-M.); k.kerrigan@sheffield.ac.uk (K.K.); 2 Industrial Doctorate Centre in Machining Science, The University of Sheffield, Sir Frederick Mappin Building, Mappin Street, Sheffield S1 3JD, UK; 3 Department of Mechanical Engineering, The University of Sheffield, Sir Frederick Mappin Building, Mappin Street, Sheffield S1 3JD, UK; p.fairclough@sheffield.ac.uk

**Keywords:** carbon fibre, machining, fiction, tribology, computational modelling

## Abstract

Constant coefficients of friction (COFs) are currently used in the literature to describe the contact mechanics between tool and workpiece for finite element (FE) machining simulation of carbon fibre-reinforced polymers (CFRPs). However, these are solely based on closed-loop tribology experimentation, which insufficiently represent machining conditions. To overcome this gap in the knowledge, this work proposes a novel experimental open-loop tribological testing method to produce a dynamic FE friction model for CFRP machining simulations. The newly proposed dynamic friction model is based on a function of fibre angle, contact pressure and slip rate, and it has been validated to both experimental results and constant COF FE simulations. The main aim of this article is to create a link between machining, tribology and FE simulation, by implementing cutting-edge tribological testing that results in highly accurate FE simulations. This dynamic model has been shown to improve the accuracy of open-loop tribological simulations, giving confidence in future implantation in CFRP machining simulations.

## 1. Introduction

**Figure 1:**
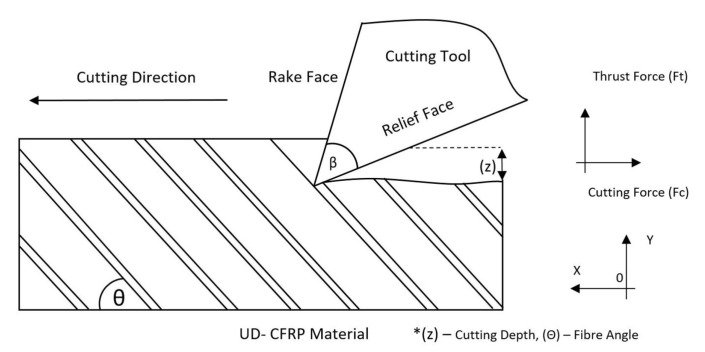
CFRP orthogonal cutting diagram with forces.

Carbon fibre-reinforced polymers (CFRPs) are being increasingly adopted in many engineering applications. CFRPs’ unique ability to be manufactured to near net shape, vast weight savings, increased strength and resistance to fatigue and corrosion make them the obvious choice for aerospace, automotive and renewables. However, the fundamental contact mechanics that occurs in machining is still relatively undefined. These tool–workpiece interactions give rise to extremely high pressures, temperatures and slip rates, thus affecting the machined surface and further affecting the contact between tool and surface. Measurement and quantification of these machining variables are extremely difficult to do experimentally, due to the inaccessibility of the contact region. FE simulations have the capacity to determine these otherwise unknown variables and graphically represent the entire contact interface. Cutting and thrust force predictions are used to quantify a simulation’s accuracy and validity (Figure 1). Well-validated machining simulations can then be used to justify tool design parameters, cutting geometry (rake/relief angles) [1], coatings, coolant and machining strategies [2]. The predictive capability of a given FE simulation can therefore provide a significant benefit, both in enabling the development of advanced cutting tools and cost savings over experimental testing [3]. Orthogonal cutting simulations are where most researchers have concentrated their efforts Table 1.

Material models are extensively researched aspects of machining simulations, with developments constantly improving validation quality. These mathematical representations of a material in the numerical domain describe how the material behaves under a given force, through elastic response, damaged initiation, plastic deformation and full damage. The most renowned models for prediction of fibre and matrix response within unidirectional composite materials are the Hashin [4] and Puck [5] models. These models are material dependent and many properties have to be tested experimentally to increase simulation validity. Recent developments in material modelling for CFRPs include linear damage propagation, calculated using the material’s fracture energy [1]. This is much more representative than purely damage initiation and element deletion methods previously proposed [6]. Similarly, friction models and COF values have been shown to greatly affect the accuracy of cutting force measurements of machining simulations [7,8]. Apparent COF (ACOF) values and material-linked friction models in metal cutting simulations have been studied more extensively than CFRP materials [9]. Due to the lack of stick–slip contact mechanism in CFRP machining, chip formation characteristics and the complex anisotropic material structure of CFRPs, no comparisons can be made between metallic and composite machining approaches or simulation strategies [10]. Frictional effects on secondary valuation criteria, such as temperature and frictional energy in CFRPs, have been known to be an area of concern for many years [11]. The simplest description for a mathematical model of the frictional forces on a macroscopic scale is the Coulomb law, which states the ratio of tangential force $F_{t}$ and normal forces $F_{n}$ [12]. This relationship gives the proportionality between the forces and the friction coefficient.

(1) $$\mu_{app}=\frac{F_{t}}{F_{n}}$$

Frictional effects can be separated into two constitute parts, an adhesive term and a deformative term [12]. When using frictional data with finite element (FE) simulation, the adhesive friction coefficient (ADCOF) should be used, as the FE solver computes the contact’s deformative friction coefficient (DCOF) from the deformation of the domain’s elements. This is an aspect that has been overlooked by a number of researchers in previous studies, as shown in Figure 1.

(2) $$\mu_{ACOF}=\frac{F_{t}}{F_{n}}=\mu_{ADCOF}+\mu_{DCOF}$$

The most common method of implementing frictional data is achieved by applying a constant Coulomb fiction coefficient value. When doing this, the user restricts the model’s ability to capture interdependencies, which are known to affect machining forces such as fibre angle (θ), pressure (*p*), slip rate (*V*), temperature (*T*) and tool edge radius (*r*). Therefore, the ACOF should be represented as a function of these variables.

(3) $$\mu_{app}\left(ACOF\right)=f\left(\theta ,p,V,T,r\right)$$

In order to account for these factors, their effects have to be isolated and quantified. Friction coefficient identification and testing have been a keen area of research, with many testing methods developed. Traditionally, to assess a pair of materials’ ACOF, a closed-loop tribometer would be used, such as a pin on disk. This apparatus involves a rotation disk of one material and a pin on a predetermined size of another material. The pin is then pressed into the disk with a given force determined by a load cell, and with a ratio of forces, the ACOF is then calculated.

**Figure 2:**
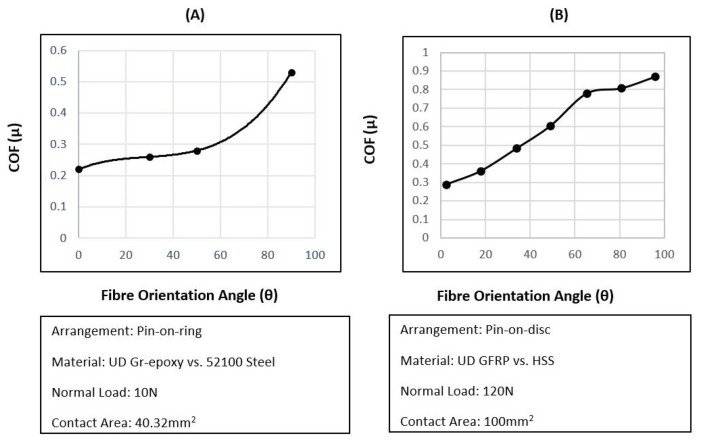
ACOF as a function of fibre angle [13,14].

There are many disadvantages to this approach when using the calculated ACOF for a machining simulation. The  maximum capable contact force and slip rate are far lower than those present in an industrial machining operation. Another factor which is not representative of machining is the wear track, as the pin drags over the same wear track throughout the trial. Inherently, machining is a material removal process, which means that fresh material comes into contract with the cutting tool at all times. Sung et al. and Nayak et al. are the only notable closed-loop tribological studies of CFRPs, showing the effects of COF and fibre angle [13,14]. Although these studies were investigating the effects of glass fibre-reinforced polymers (GFRPs) and high-speed steel (HSS) on ACOF it can be seen from Figure 2 that ACOF is clearly affected by fibre angle.

Table 1 highlights the difference in (CFRP/WC) ACOF values used throughout FE orthogonal cutting simulations carried out in previous studies. The mentioned authors have referenced the pin and disk studies previously discussed as their source for ACOF values. Others have chosen to take an average of previous cited ACOF values [15]. Some have even discussed using various ACOF values to match force responses with experimental data [11,16].

Friction separation methods have been overlooked by all the studies motioned above, with the apparent COF (ACOF) as the value used in simulations instead of ADCOF values. One analytical method of achieving friction separation was developed by Challen et al. and more recently was implemented by Mondelin et al. for machining of CFRPs [23,24]. This ploughing model showed promising results for metal ploughing; however, this utilises a method developed by Johnson and Rowe [23] for standing wave theory. A few assumptions are needed for this elastic recovery model to be valid; one notable assumption is that no material spring back is considered.

This assumption was justified by Mondelin et al. due to the fact that CFRPs have a low Young’s Modulus and the effects of normal force and therefore the contact pressure on results, so neglecting spring back effects was thought to be adequate. However, it has been shown by preliminary cutting trails that for orthogonal cutting, the spring-back for a 200 μm depth of cut can be in the region of 75 μm [25]. Another analytical model proposed by Lafaye et al. is considered to be more appropriate as it is designed for a spherical tipped pin on a material with elastic recovery. This model was used to good effect by Klinkova et al. to develop a velocity-dependent coefficient of friction for machining CFRPs [26]. Although a good improvement upon the purely plastic model used by Mondelin et al., analytical models are known to have high sources of error for this reason, and therefore a novel method has been proposed, using a numerical simulation to inversely separate the frictional COFs.

**Figure 3:**
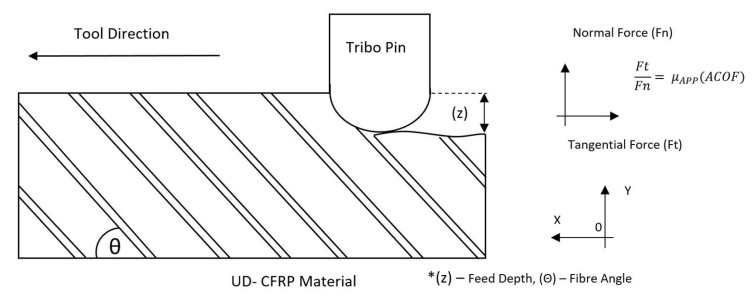
CFRP open-loop tribometer diagram with forces.

A number of more recent studies have tried to close the gap between the use of these outdated pin of disk trials and shine a light on the misuse of ACOF values in FE machining simulations. Open-loop tribometers are thought to be the cutting edge of tribological study for machining applications—these systems take advantage of highly accurate machining centres equipped with a pin of predetermined geometry and piezoelectric dynamometer to collect forces. This allows the user to design a tribological test that best represents the machining situation that is being investigated, with much higher forces, speeds and presentation of fresh material to the workpeice—a vast improvement upon the closed-loop systems. Figure 3 demonstrates how the forces from the dynamometer are used to calculate the material pair’s ACOF. Comparing this diagram to the one of orthogonal cutting (Figure 1), major similarities can be drawn. By replacing the cutting tool for a pin, the cutting forces are isolated from the contact, leaving only the ploughing effect—vastly decreasing the complexity of the system, thus allowing for the frictional forces to separated from the cutting forces as best as possible.

**Figure 4:**
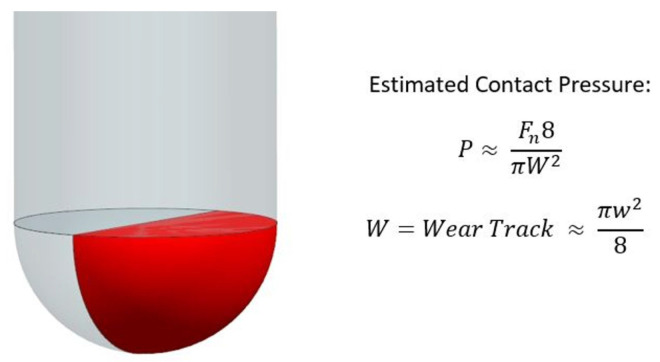
Geometric approximation for pressure proposed by Zemzemi [28] and implemented by Mondlein [24].

Constant sampling of these forces allows for dynamic friction models to be developed, in which friction is not a contact value but a function of a number of other variables (Equation (3)). Mondelin et al.’s novel approach using an open-loop tribometer with CFRPs highlighted that apparent COF can be sliding velocity dependent, and was a first of its kind [24]. Pressure was not seen to influence apparent COF response, although this is thought to be due to the use of the Zemzemi approximation (Figure 4) with composite materials. Due to the high levels of material spring-back in CFRPs, measuring the wear scar after the contact has occurred to calculate contact pressure is thought to be full of error and uncertainty. Klinkova et al. and Chardon et al. showed that apparent COF can be affected by sliding velocity in short fibre composite materials [26,27]. Voss et al. indicated that roughness and fibre angle also affect ACOF. All findings are intuitive and realistic, further displaying the oversimplification of the use of a constant COF in any FE cutting simulation. Xu et al. carried out both an experimental and a numerical study into the apparent COF effects for CFRPs. Normal force was shown to affect the apparent COF with an exponential decay and a steady-state region after 100 N and up to 250 N. However, using normal force to derive a dynamic ACOF is case specific. Depending on the pin size used, the force response will be different, with the only method to overcome this being using pressure to normalise the results. The material model used in this study is the maximum stress model with no damage propagation, which is a relatively basic approximation of the material response. Given the previous work in the field, the main aims for this study are to successfully develop an open-loop tribometer, in order to develop a dynamic friction model for CFRPs that is a function of slip rate, fibre angle and pressure.

## 2. Experimental Work

### 2.1. Setup and Procedure

**Figure 5:**
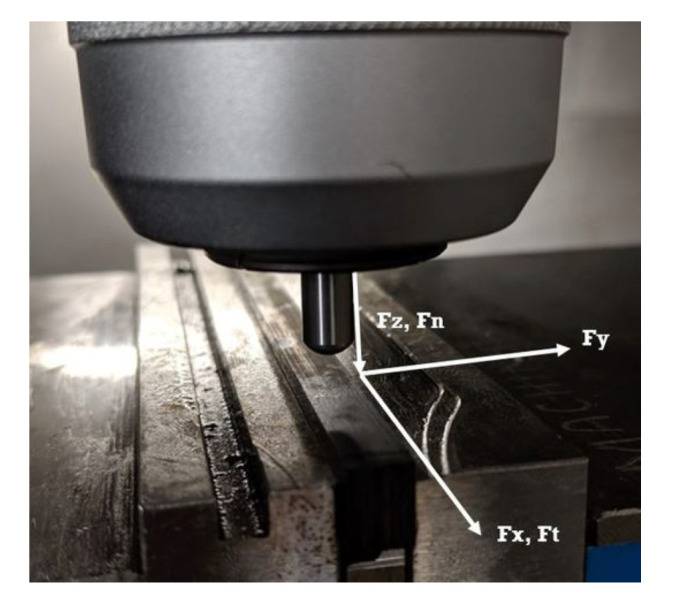
Axis of force direction.

A DMG Evo 40 linear 5-axis machine and A Kistler (9139AA) compact multi-component dynamometer were used along with a Kistler charge amplifier, which is capable of reading forces up to 30 kN and a sampling rate of 1 MHz. Samples of UD-CFRP with Toray T700SC fibres and a XPREG XC130 resin system were manufactured to 150 × 50 × 6 mm with fibre angles of 22.5 to 157.5 in increments of 22.5. These were aligned though the x, z plane with 0 were placed on the x: (1,0) a tool moving from x: (−1) to x: (1) Figure 5.

A WC uncoated pin with a radius of 3.25 mm was used to contact with the CFRP surface. Indent depths were at 100 μm and 250 μm, and used along with slip rates of 1 to 15 m/min, increasing in 5 m/min increments.

Pressure measurements were post processed by assuming the machine tool was perfectly accurate and stiff, which was possible because the in feed (Z) was controlled throughout the trial. This then allowed the use of a geometric relationship of a sphere, with (z) as the feed depth and (r) as the pin radius in Equation (4).

(4) $$\begin{array}{c} A=2\pi rz\\ P=f_{n}/A \end{array}$$

### 2.2. Experimental Results

**Figure 6:**
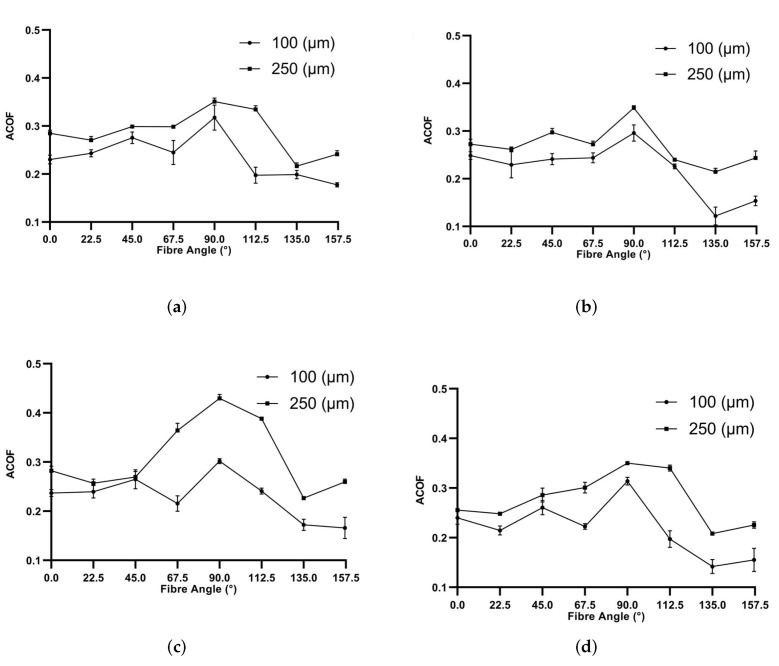
Effects of fibre angle on ACOF with machining velocities: (**a**) 1 m/min, (**b**) 5 m/min, (**c**) 10 m/min and (**d**) 15 m/min.

Fibre angle, contact pressure and slip rate-dependent ACOF data are displayed in Figure 6. The effects of each variable are difficult to quantify in (x,y) plots, because of the interdependence between the variables. For this reason, multi-linear regression (MLR) has been carried out to develop an empirical approximation of the factors. However, in more qualitative terms, a definite change of ACOF as an effect on fibre angle can be seen, with 90 θ resulting in the highest ACOF value, which agrees with both closed-loop studies previously discussed [13,14]. Effects of feed and therefore pressure are also apparent—higher feeds yield higher ACOF values, which was expected.

The effects of sliding speed are more difficult to see, showing a minimal effect on ACOF—this is most likely due to a limitation of the maximum available sliding speed 15 m/min. Higher speeds of 50 m/min were achievable with the current setup, although at a cost to the machine’s accuracy in both feed and speed. Further work is needed in this area with the possible inclusion of a CNC Lathe instead of a 5 axis machining centre to achieve accurate speeds of 500 m/min.

A multi-linear regression (MLR) algorithm was used to empirically fit the experimental ACOF data. The MLR was carried out using MATLAB’s ‘regress’ function—this allowed for a dynamic APP COF to be created with respect to a given fibre angle ($F_{i}$), pressure ($P_{i}$) and cutting speed ($V_{i}$). Table 2 gives insight into the dominance of each factor, with pressure and fibre angle being by far the most dominant factors, which agrees with the graphs above. The quality of fit in general was acceptable, with an R2 of 0.789. Velocity has been shown to have minimal effects on ACOF within the 1 to 15 m/min range; however, this does not represent the effects at much higher speeds.

(5) $$\mu_{Adhesive}=\beta_{0}+\beta_{1}\left(V_{i}\right)+\beta_{2}\left(P_{i}\right)$$

## 3. Numerical Work

### 3.1. Introduction

This section will discuss the issues faced when implementing dynamic experimental frictional data—collected from the previous experimental section—into an explicit machining simulation though the use of ABAQUS CAE finite element solver. Dynamic friction coefficients are not possible in ABAQUS CAE GUI, and this section will discuss how this problem was overcome. The main advantage of initially using FE is to allow for a more accurate method of friction serration. As previously mentioned, ABAQUS/CAE by default only requires ADCOF as an input for the contact properties, meaning that the ACOF data from the experimental work needs to be separated—this was achieved by using an inverse modelling technique. Once the data had been separated, MLR was then carried out in the ADCOF data to build a dynamic friction model for WC-CFRPs.

Another key factor of ABAQUS CAE is that the built-in Coulomb friction model does not depend on any factors, apart from a constant value widely accepted at approximately 0.3 for CFRP/WC variables—this is a major assumption in previous numerical studies. CFRPs also have the added difficulty of having a fibre orientation-dependent coefficient of friction, further highlighting the need for a detailed friction model to remove key uncertainties in FE modelling of CFRP machining operations.

**Figure 7:**
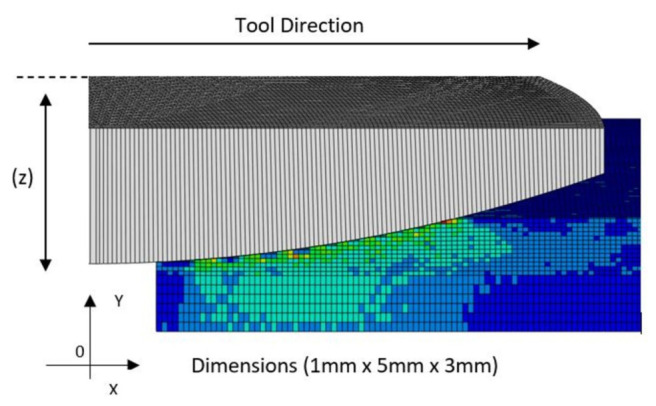
2D Cross section of the 3D FE model.

The final separated ADCOF data were then applied though a user-defined subroutine, in a number of numerical tribological studies, to discuss the benefits of using the newly proposed dynamic friction model.The Finite element model used for the friction separation method can be seen in Figure 7.

### 3.2. Material Model

In the public domain, high-quality material behaviour models are essential to perform accurate simulations. The greatest complexity in material models is usually found in the design of reliable damage models. In the case of composite materials, the development of a damage model is complex due to the interaction of fibre and matrix damage types. This work would complicate this research focused on the study of tool/workpiece friction. Therefore, this work implements the model implemented by Cepero-Mejias et al. in various fields of composite machining modelling such as chip formation [29,30], tool wear [3] and sub-surface damage [1,31,32] to obtain high-quality predictions.

This model consists of an orthotropic linear-elastic model before any initiation of damage occurs. Damage initiation is calculated using a Hashin–Puck hybrid model considering four types of damage: fibre traction (ft), fibre compression (fc), matrix traction in ply (mt2), matrix compression in ply (mc2), matrix traction in the thickness direction (mt3) and matrix compression in the thickness direction (mc3). Exposure factors ($F_{I}$) control damage initiation with the equations presented below.

- Fibre traction $\left(\sigma_{11}\geq 0\right)$
  (6) $$F_{ft}={\left(\frac{\sigma_{11}}{X_{T}}\right)}^{2}+{\left(\frac{\sigma_{12}}{S_{12}}\right)}^{2}+{\left(\frac{\sigma_{13}}{S_{13}}\right)}^{2}\geq 1$$
- Fibre compression $\left(\sigma_{11}<0\right)$
  (7) $$F_{fc}=|\frac{\sigma_{11}}{X_{C}}|\geq 1$$

- Matrix Mode A in ply $\left(\sigma_{22}\geq 0\right)$
  (8) $$F_{mt2a}=\sqrt{{\left(\frac{\sigma_{12}}{R_{⊥\|}^{A}}\right)}^{2}+{\left(1-\frac{p_{⊥\|}^{\left(+\right)}}{R_{⊥\|}^{A}}R_{⊥}^{(+)A}\right)}^{2}{\left(\frac{\sigma_{22}}{R_{⊥}^{(+)A}}\right)}^{2}}+\frac{p_{⊥\|}^{\left(+\right)}}{R_{⊥\|}^{A}}\sigma_{22}\geq 1$$
- Matrix Mode B in ply $\left(\sigma_{22}<0\;and\;\sigma_{22}>-R_{⊥⊥}^{A}\right)$
  (9) $$F_{mc2b}=\sqrt{{\left(\frac{\sigma_{12}}{R_{⊥\|}^{A}}\right)}^{2}+{\left(\frac{p}{R}\right)}^{2}\sigma_{22}^{2}}+\left(\frac{p}{R}\right)\sigma_{22}\geq 1$$
- Matrix Mode C in ply $\left(\sigma_{22}\leq -R_{⊥⊥}^{A}\right)$
  (10) $$F_{mc2c}=\frac{1}{2\left[1+\left(\frac{p}{R}\right)R_{⊥⊥}^{A}\right]}\left[{\left(\frac{\sigma_{12}}{R_{⊥\|}^{A}}\right)}^{2}+{\left(\frac{\sigma_{22}}{R_{⊥⊥}^{A}}\right)}^{2}\right]\frac{R_{⊥⊥}^{A}}{-\sigma_{22}}\geq 1$$

- Matrix traction in thickness direction $\left(\sigma_{33}\geq 0\right)$
  (11) $$F_{mt3}={\left(\frac{\sigma_{33}}{Z_{T}}\right)}^{2}+{\left(\frac{\sigma_{13}}{S_{13}}\right)}^{2}+{\left(\frac{\sigma_{23}}{S_{23}}\right)}^{2}\geq 1$$
- Matrix compression in thickness direction $\left(\sigma_{33}<0\right)$
  (12) $$F_{mc3}=|\frac{\sigma_{33}}{Z_{C}}|\geq 1$$

For brevity, the meaning of the terms stated in the above equations is not explained in this manuscript. The reader is referred to [5] for more information on the terms used in these equations. Once damage initiation occurs for a certain damage mode, a linear degradation based on energy criteria is applied to the stiffness matrix components associated with the matrix or fibre. This action is achieved by introducing matrix damage ($d_{m}$), fibre damage ($d_{f}$) and shear damage ($d_{s}$) variables in the components that affect longitudinal, transverse or shear stiffness, as shown in Equation (13).

(13) $$\begin{array}{c} C_{11}=E_{11}\left(1-d_{f}\right)\left[1-\left(1-d_{m2}\right)\left(1-d_{m3}\right)\nu_{23}^{2}\right]/A\\ C_{12}=E_{22}\left(1-d_{f}\right)\left(1-d_{m2}\right)\left[\left(1-d_{m3}\right)\nu_{13}\nu_{23}+\nu_{12}\right]/A\\ C_{22}=E_{22}\left(1-d_{m2}\right)\left[1-\left(1-d_{f}\right)\left(1-d_{m3}\right)\nu_{13}\nu_{13}\right]/A\\ C_{13}=E_{33}\left(1-d_{f}\right)\left(1-d_{m3}\right)\left[\left(1-d_{m2}\right)\nu_{12}\nu_{23}+\nu_{13}\right]/A\\ C_{33}=E_{33}\left(1-d_{m3}\right)\left[1-\left(1-d_{f}\right)\left(1-d_{m2}\right)\nu_{12}\nu_{21}\right]/A\\ C_{23}=E_{33}\left(1-d_{m2}\right)\left(1-d_{m3}\right)\left[\left(1-d_{f}\right)\nu_{12}\nu_{31}+\nu_{23}\right]/A\\ C_{44}=G_{12}\left(1-d_{f}\right)\left(1-d_{m2}\right)\\ C_{55}=G_{13}\left(1-d_{f}\right)\left(1-d_{m3}\right)\\ C_{66}=G_{23}\left(1-d_{m2}\right)\left(1-d_{m3}\right) \end{array}$$

Here, $\;d_{f}=max\left\{d_{ft},d_{fc}\right\}\;;\;d_{m2}=max\left\{d_{mt2},d_{mc2}\right\}\;;\;d_{m3}=max\left\{d_{mt3},d_{mc3}\right\}$ $d_{I}\epsilon \left[0,1\right]\;$ and I=(ft,fc,mt2,mc2,mt3,mc3) with $\;A=1-\left(1-d_{f}\right)\left(1-d_{m2}\right)\nu 12\nu_{21}-\left(1-d_{m2}\right)\left(1-d_{m3}\right)\nu_{23}^{2}-\left(1-d_{f}\right)\left(1-d_{m3}\right)\nu_{13}\nu_{31}$ $-2\left(1-d_{f}\right)\left(1-d_{m2}\right)\left(1-d_{m3}\right)\nu_{12}\nu_{31}\nu_{23}$.

These damage variables evolve from 0 (no damage) to 1 (total damage), guaranteeing a linear degradation of the mechanical properties of each element until the damage mode fracture energy is reached. Note that the maximum matrix damage is set to 0.95 to avoid distortional problems [19] and consider the remaining stiffness that a cracked matrix ply brings to adjacent plies [33]. This degradation of the mechanical properties is carried out between an initial equivalent displacement value ($\delta_{I,eq}^{0}$) and a final equivalent displacement value ($\delta_{I,eq}^{f}$), calculated with Equations (14) and (15). Finally, Table 3 shows the fracture energies of each damage mode used in this investigation.

(14) $$\delta_{I,eq}^{0}=\frac{\delta_{I,eq}}{F_{I}}$$

(15) $$\delta_{I,eq}^{f}=\frac{2G_{I}^{C}}{\sigma_{I,eq}}$$

(16) $$d_{I}=\frac{\delta_{I,eq}^{f}\left(\delta_{I,eq}-\delta_{I,eq}^{0}\right)}{\delta_{I,eq}\left(\delta_{I,eq}^{f}-\delta_{I,eq}^{0}\right)}\;\left(d_{I}\in \left[0,1\right]\;and\;I=\left(ft,fc,mt2,mc2,mt3\;and\;mc3\right)\right)$$

### 3.3. Inverse Modelling Friction Separation

The experimental apparatus was modelled in an ABAQUS FE explicit domain, a mesh sensitivity study was undertaken and a 5 µm C3D8R element size was used. A hard penalty contact algorithm was selected along with a rigid boundary condition for the pin. A material percentage damage to failure of 95% was used, which is a regularly accepted figure in the literature [19].

**Figure 8:**
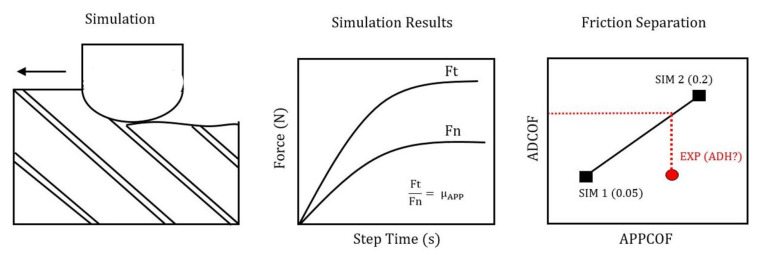
Friction separation flowchart.

A linear response was observed between ACOF and ADCOF, which was expected due to the way in which the Coulomb friction model is implemented in ABAQUS. This greatly reduces the number of iterations needed in the inverse modelling of the friction separation Figure 8. Separation was achieved by inputting ADCOF into the open-loop tribo simulation through a standard Coulomb friction model. Values 0.05 and 0.2 were used; however, due to the linear response, any values within the range could have been used. Once the global forces stabilised, ACOF could be calculated. Knowing the ACOF and ADCOF from each simulation and the ACOF from the experimental results, the experimental ADCOF could be established.

The separation ratios were then applied to the ACOF MLR data to create ADCOF MLR data to be used in further FE simulations, shown in Table 4. Due to the complex physics which accrue throughout different fibre angles, buckling, debonding, and tearing, ADHCOF has been separated into independent fibre angle MRL data—this greatly improved its accuracy.

(17) $$\mu_{Adhesive}=(\beta_{0}+\beta_{1}\left(V_{i}\right)+\beta_{2}\left(P_{i}\right)$$

### 3.4. Finite Element and Friction Modelling

During a machining FE simulation, when the tool and workpiece come into contact, an algorithm is used to determine how the nodes and nodal surfaces interact with one another. During this stage, a mechanical constrained formation is applied, either a penalty or kinematic contact method, in which local contract pressures, sliding velocities and temperatures are calculated.

These contact variables can then be supplied to the frictional model, meaning that the contact algorithm is only physically representative if the fictional model is accurate.

### 3.5. Empirical Friction Model

A dynamic friction model for WC-CO and UD CFRPs, which is a function of fibre orientation, sliding velocity and applied force, is the proposed solution to uncertainty present when using a contact COULOMB model. This empirical solution has been implemented using a user-defined subroutine, which does not remove workload from the FE solver, as the material model and contact algorithm would still need to compute the normal forces. Even so, the dynamic frictional changes and their effect on first-order and secondary results are better accounted for.

### 3.6. GUI Tangential Behaviour

The simplest method which has previously been discussed is to apply a constant friction coefficient through the contact property in the GUI in ABAQUS. This method has the benefit of being robust and simple to apply, but does not capture dynamic frictional changes. This method also has the ability to use slip rate-, contact pressure- and temperature-dependent data, which is a great asset for the GUI program. However, these data are applied in a spreadsheet format and do not allow for a single empirical model to be applied.

### 3.7. VFRIC and VFRICTION Subroutine

VFRIC user subroutine can be used to define frictional behaviour between a contact pair of surfaces, when the typical Coulomb model is too restrictive and a more complex shear transition between the surfaces is present. VFRIC can be used to control other solution-dependent state variables, and would allow for a single constitutive friction model to be applied, unlike the GUI format. However, the main disadvantage of this subroutine is that it can only be used with the `surface to surface’ contact algorithm and not with `general contact’ (GC). Surface to surface also has the issue of self intersecting internal nodes during the simulation, which can affect the simulation’s ability to complete without computational errors.

VFRICTION is very similar to VFRIC, and is programmed in the same manner but has fewer variables that it can control and can be used with the GC algorithm. The GC algorithm is thought to yield better results for machining simulations than the surface-to-surface contact algorithm, although it can only do so in a 3D case, which greatly increases the computational time. However, pressure is a 3D phenomenon and for this reason, VFRICTION was the chosen subroutine for this study. A 3D benchmark was carried out against ABAQUS/CAE built-in Coulomb friction and VFRICTION, in which complete correlation was achieved, giving good confidence in the VFRICTON subroutine.

## 4. Numerical Pressure Measurements and Conversion

The final goal of this numerical section is to achieve a pressure-, speed- and fibre angle-driven friction model, which works on a nodal scale developed with experimental factors on a global scale—meaning a method of measuring pressure on an experimental level which correlates with pressures measured on a nodal scale is imperative. Experimental pressure was calculated as previously discussed (Equation (18)) using the global load on the pin and the feed depth. To ensure that the nodal pressures and this global pressure were valid, a benchmark was undertaken. Nodal pressure was outputted from the VFRICTION subroutine Equation (19), with fNormal(K) being the nodal force and AreaCont(K) equalling the instantaneous area in contact. CPress is an inbuilt command in ABAQUS/CAE, which is calculated using the net normal force loads, CNORNF and the element area in contact with the pin’s surface.

(18) $$\begin{array}{c} ContactArea(A)=2\pi rz\\ GlobalPressure=f_{n}/A \end{array}$$

(19) $$NodalPressure\left(K\right)=\frac{fNormal(K)}{AreaCont(K)}$$

**Figure 9:**
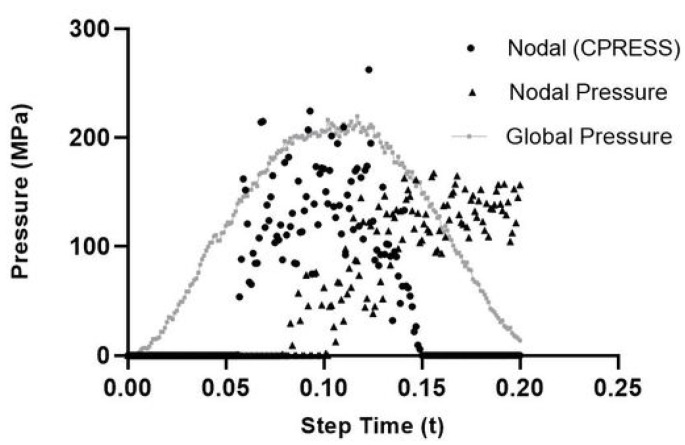
CPress, NPress and GPress comparison.

Figure 9 shows a scattering of pressures for both nodal pressure measurements. This is to be expected as these instantaneous values of pressures are constantly changing throughout the simulation. This is due to the material model’s response and its resulting force (fn) on the pin. What is more interesting is the scale of forces, with both global and nodal methods peaking at 200 MPa, which gives confidence overall in using this method and in Equation (18).

## 5. Dynamic Friction Implementation

**Figure 10:**
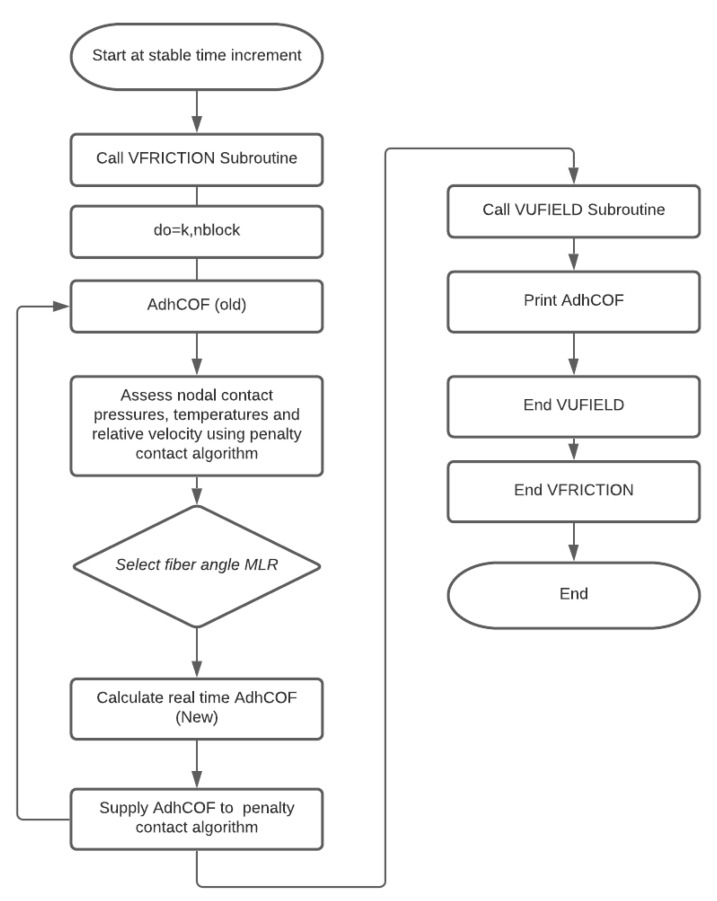
Subroutine implementation.

Using the adhesive friction model developed using the friction separation method previously discussed, a number of validation simulations were tested. This was to demonstrate the robustness and accuracy of the newly proposed dynamic friction model. Figure 10 shows the order in which the VFRICTION subroutine (Listing 1) is computed, which is called at every numerical interval and iterated throughout the FE simulation. Incorporating the required subroutines (VUMAT, VFRICTION, and VUFIELD) with multi-thread computation, this was achieved using common blocks. Using mpi threads did not require a locking and unlocking function in order for the computation to work correctly [34].

**Listing 1:**
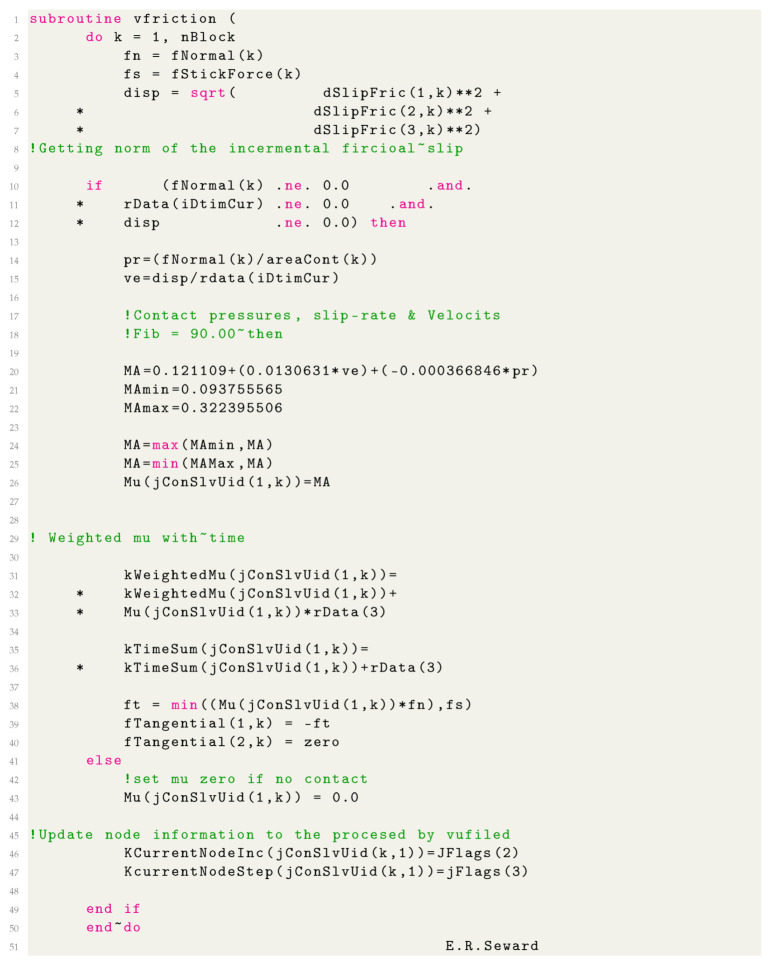
Subroutine code.

## 6. Results and Discussion

**Figure 11:**
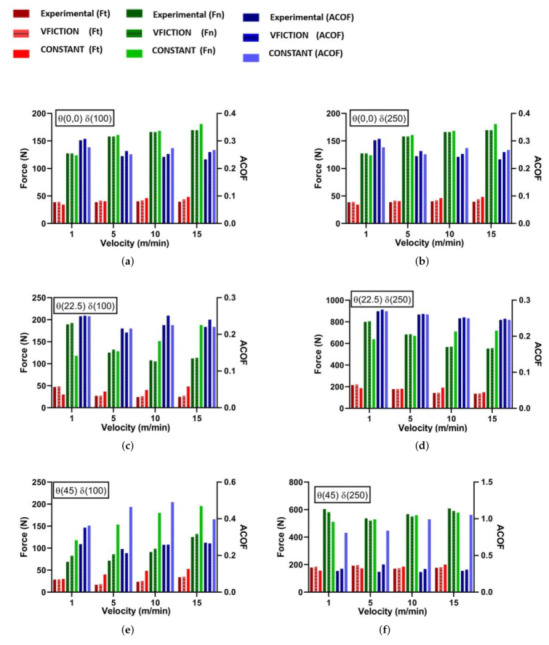
Experimental Results: FE Comparison of Dynamic Friction Model vs. Constant Friction Coefficient for Open-Loop Tribology.

**Figure 12:**
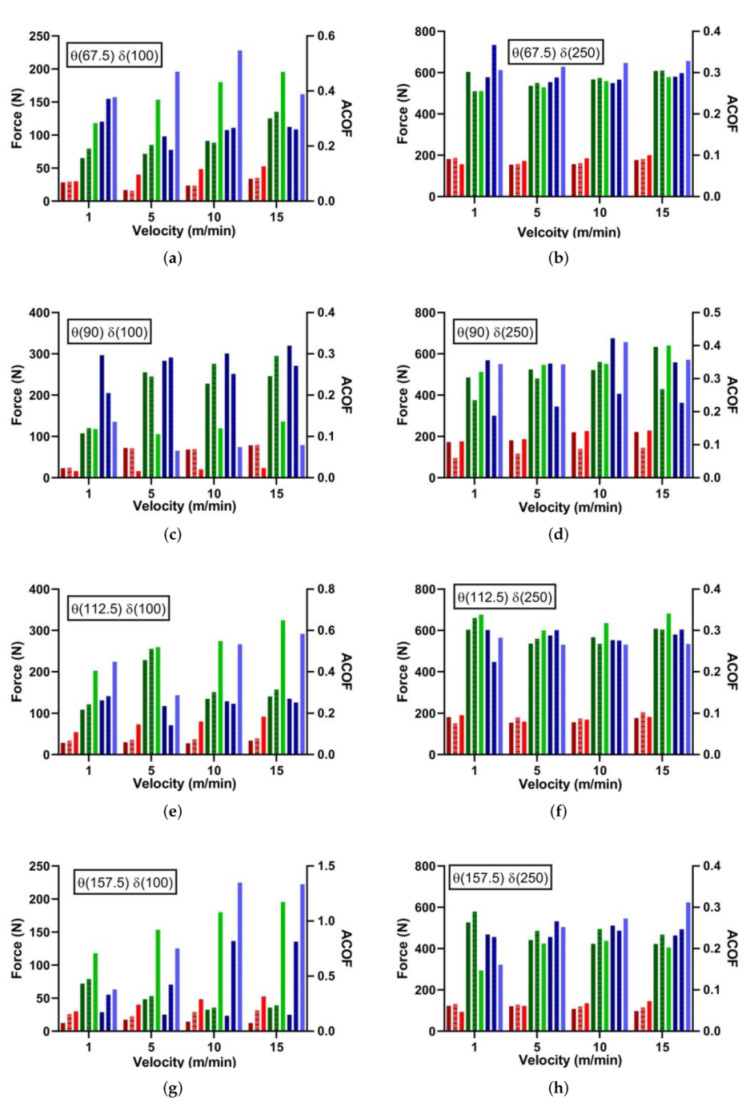
Experimental Results: FE Comparison of Dynamic Friction Model vs. Constant Friction Coefficient for Open-Loop Tribology.

All open-loop tribo interactions were simulated using the same methodology previously discussed, although the dynamic friction model was implemented through the VFRICTION subroutine for these simulations. Comparing VFRICTION, constant and experimental forces and ACOF show good correlation (Table 5, Figure 11 and Figure 12). Positive fibre angels (0–90) show the least initial error with 0.2 AHCOF performing relatively well. However, VFRICTION still gave improvements of 1%, 13% and 12% for Fn, Ft and ACOF, respectively. Negative fibre angles showed the highest improvements, of 18%, 18% and 26% for Fn, Ft and ACOF respectively. Negative angles are predominantly harder to model due to the increased complexity in failure modes and chip formation, which causes numerical instability. Therefore, the results collected using VFRICTION at these angles are promising for the methodology. However, some caution should be taken with this set as the R2 values for θ (90 and 112.5) were relatively low due to experimental difficulties when machining these negative angles.

**Figure 13:**
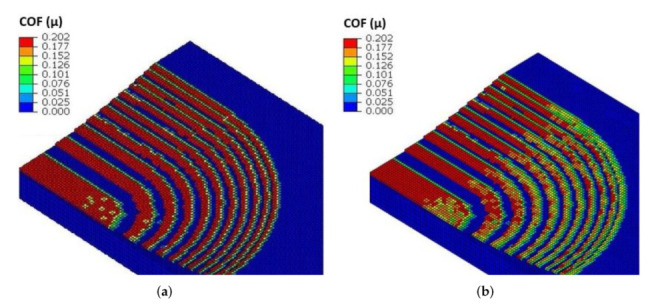
A section of the tribo open-loop FE model with the pin removed—constant (**a**); VFRICTION (**b**).

Benefits of using the VFRICTION subroutine over a constant ACOF can be seen below, with VFRICTION allowing dynamic friction across the surface in contact, unlike its constant counterpart, which is thought to be more physiologically accurate to the real-word situation. The constant friction model only allows for a step function (0–0.2) ADCOF when the elements of the pin and CFRP come into contact. In comparison, VFRICTION allows a dynamic function of ADCOF driven with data collected from the experiment, which can be seen graphically bellow in Figure 13b.

Using this methodology removes the uncertainty and ability to adjust COF values to match up real-world machining forces. However, it does further complicate CFRP machining simulations and requires researchers to carry out not only material testing but also open-loop tribological testing before developing FE simulations.

## 7. Conclusions

This study has shown the current uncertainty in literature found when constant friction coefficients are implemented for FE machining simulations, whilst highlighting the benefits of using a dynamic friction model, to capture in more detail the factors that affect the tool–workpiece interaction. To the authors’ knowledge, no other study is yet to link open-loop tribological data for CFRPs, with a well-validated FE material model. Further improvements could be made with the available dataset, and a higher number of feeds, speeds, and fibre angles could be tested. Further, differing CFRP compositions and temperature effects would improve the validity of the proposed friction model, although the experimental open-loop tribology setup, FE modelling methodology, and dynamic friction model implementation have set the framework for further study in the sector.

- This novel CFRP/WC dynamic frictional model and novel implementation yielded a more accurate force response for each fibre angle studied.
- The dynamic friction model was shown to over predict 2% at 100 μm and under predict 4% at 250 μm, which should improve with more data to the tune friction model, compared with an average 10% when using a constant value.
- Velocity changes have a much greater effect on the contact friction model than pressure.
- A limitation of this study is that it does not account for temperature effects in both the experimental trial and numerical domain.
- 90° showed the least comparison for both dynamic and constant approaches.

## Figures and Tables

**Table 1:** Orthogonal cutting COF comparison table for previous simulations.

Authors	Publication Date	ACOF Used
Ramulu et al. [17]	1997	0.4
Arola et al. [18]	2002	0.4
Bhatnagar et al. [16]	2014	N/A
Mkaddem et al. [15]	2008	0.3
Lasri et al. [19]	2009	0.5
Santiuste et al. [11]	2010	0.5
Santiuste et al. [20]	2011	0.5
Zenia et al. [21]	2015	0.4
Benhassine et al. [22]	2018	0.3
Cepero et al. [1]	2019	0.2

**Table 2:** MLR Coefficients.

Θ	$\beta_{0}$	$\beta_{1}$	$v\beta_{2}$	R2
0	0.2803690	−0.0015720	−0.0012253	0.93
22.5	0.1644200	0.0050902	−0.0006417	0.837
45	0.0974485	−0.0004083	0.0001334	0.905
67.5	0.3047650	−0.0053778	−0.0006111	0.905
90	0.1211090	0.0130631	−0.0003668	0.738
112.5	0.1763290	−0.0039313	0.0008020	0.679
135	0.1679290	−0.0069309	0.0006020	0.837
157.5	0.1060290	0.0018082	0.0007306	0.949

**Table 3:** Critical fracture toughness.

N/mm	$G_{\mathrm{ft}}^{c}$	$G_{\mathrm{fc}}^{c}$	$G_{\mathrm{mt}2}^{c}$	$G_{\mathrm{mc}2}^{c}$	$G_{\mathrm{mt}3}^{c}$	$G_{\mathrm{mc}3}^{c}$
$G_{I}^{C}$	100	100	1	1	1	1

**Table 4:** MLR Data.

Θ	$\beta_{0}$	$\beta_{1}$	$\beta_{2}$	R2
0	0.052488	0.006025	0.0003908	0.847
22.5	0.197959	−0.001155	−0.000634	0.793
45	0.240492	−0.005277	−0.000341	0.786
67.5	0.18297	0.007671	−0.000392	0.466
90	0.149911	0.013133	−0.000753	0.715
112.5	0.081576	−0.001422	0.0008751	0.634
135	0.156666	0.014112	−0.000684	0.689
157.5	0.075583	0.000894	0.000419	0.7111

**Table 5:** Percentage difference for constant ACOF vs. proposed dynamic friction model.

θ	FN VFRI	FN CON	FT VFRI	FT CON	ACOF VFRIC	ACOF CON
0	−5%	0%	−2%	−8%	−5%	−8%
22.5	1%	−3%	−3%	−10%	−2%	−9%
45	4%	−19%	−4%	−18%	−9%	−54%
67.5	4%	−19%	−4%	−18%	−9%	−54%
90	7%	48%	−2%	138%	11%	166%
112.5	8%	−22%	−12%	−32%	9%	−19%
135	12%	−39%	−35%	−61%	−6%	−53%
157.5	10%	−23%	−28%	−35%	−36%	−39%
Ranges						
0–90	2%	1%	−3%	16%	−2%	14%
112.5–157	10%	−28%	−25%	−43%	−11%	−37%

## Data Availability

The data presented in this study are available on request from the corresponding author.
